# Real-world use of adjuvant nivolumab among patients with esophageal and gastroesophageal junction cancer at a large integrated health system

**DOI:** 10.1016/j.xjon.2025.10.003

**Published:** 2025-10-15

**Authors:** Andrea M. Gochi, Sydney Jeffs, Yun-Yi Hung, Jingrong Yang, Tony Wang, Gary Okano, Lisa Rosenblatt, Reginald Villacorta, Jeffrey B. Velotta

**Affiliations:** a Department of Surgery, UCSF East Bay, Oakland, Calif; b Duke University School of Medicine, Duke University, Durham, NC; c Division of Research, Kaiser Permanente Northern California, Pleasanton, Calif; d Bristol Myers Squibb Company, New York, NY; e Division of Thoracic Surgery, The Permanente Medical Group, Inc, Oakland, Calif; f Department of Surgery, UCSF, San Francisco, Calif; g Department of Clinical Science, Kaiser Permanente Bernard J. Tyson School of Medicine, Pasadena, Calif

**Keywords:** gastroesophageal junction cancer, esophageal cancer, immunotherapy, real-world evidence

## Abstract

**Objectives:**

To assess real-world use of adjuvant nivolumab in patients with esophageal/gastroesophageal junction cancer.

**Methods:**

This was a retrospective, non-interventional cohort study of electronic health record data in the Kaiser Permanente Northern California cancer registry (June 1, 2020, to October 31, 2024). Index was chemoradiation therapy date between December 1, 2020, and August 31, 2023, with 6 months baseline and ≥12 months’ follow-up. Included patients were aged ≥18 years at index; had stage II/III esophageal/gastroesophageal junction cancer; were receiving chemoradiation within the index period; had evidence of local disease after neoadjuvant chemoradiation; and were classified as Eastern Cooperative Oncology Group performance score 0-1 at index. Patient demographic and clinical characteristics, as well as clinical and patient journey outcomes, were extracted from electronic medical records, confirmed by chart review. Descriptive statistics describe outcomes with Kaplan-Meier method used for time-to-event outcomes.

**Results:**

Included patients (n = 44; median age 66.0 years; interquartile range, 61.5-73.0 years) were followed for median 19.1 months (interquartile range, 10.6-25.3 months) from nivolumab first dose. Median overall survival was not reached. The 24-month overall survival rate was 72.3% (95% confidence interval, 58.7%-89.1%). Median disease-free survival was 20.8 months, and median distant metastases-free survival was 23.7 months (95% confidence interval, 13.0-not reached). All patients underwent minimally invasive esophagectomy and had R0 resection status. Median time from index to nivolumab initiation was 165 days (interquartile range, 138.5-194.0 days). Median duration of nivolumab treatment was 322 days (interquartile range, 148.5-364.0 days); 52.3% of patients completed 1 year of nivolumab treatment.

**Conclusions:**

This study demonstrates survival benefits of adjuvant nivolumab in line with clinical studies.

**Figure:**
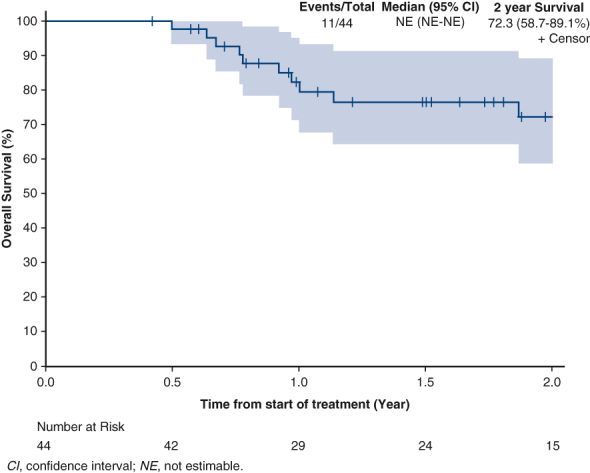
Kaplan-Meier curve of OS with adjuvant nivolumab after minimally invasive esophagectomy.

**Central Message**

Median overall survival was not reached in this cohort of resected esophageal/GEJ cancer patients treated with adjuvant nivolumab; 24-month OS was 72.3%, suggesting potential benefit.

**Perspective**

These findings, from the largest real-world cohort study of adjuvant nivolumab in patients with esophageal/gastroesophageal junction cancer to date, show that real-world outcomes in this population are similar to those seen in clinical studies. The outcomes presented may guide clinician choice and ability to estimate the patient journey in this population.

Esophageal and gastroesophageal junction (E/GEJ) cancers are classified as upper gastrointestinal tract cancers. In the United States in 2025, it is estimated that there will be approximately 22,070 new cases of esophageal cancer and 16,250 deaths.^1^ In addition, the number of GEJ cancers continues to increase.^2^^,^^3^ Previous National Comprehensive Cancer Network (NCCN) guidelines recommended chemoradiation therapy (CRT) followed by surgical resection as standard of care (SOC) for patients with early-stage E/GEJ cancer.^4^ Estimated 3- and 5-year survival rates after CRT range from 30% to 40%.^5^, ^6^, ^7^ More recently, perioperative 5-fluorouracil, leucovorin, oxaliplatin, docetaxel has emerged as an alternative SOC for resectable esophageal and GEJ adenocarcinoma, in Europe and increasingly in the United States. The phase 2/3 FLOT (5-fluorouracil, leucovorin, oxaliplatin, docetaxel-4) trial demonstrated improved overall survival (OS) compared with previous perioperative regimens such as epirubicin + cisplatin + 5-fluorouracil and epirubicin + cisplatin + capecitabine regimens, prompting wider adoption in select populations.^8^

Until 2021, although, there was no established postoperative adjuvant SOC after CRT and surgical resection. In addition, programmed cell death ligand 1 (PD-L1) expression has been reported in approximately 40% of E/GEJ cancers and is associated with a poor prognosis, including reduced OS.^7^^,^^9^ Nivolumab, a programmed cell death protein 1 checkpoint inhibitor, was the first adjuvant therapy to provide a statistically significant and clinically meaningful improvement in disease-free survival (DFS) versus placebo in resected E/GEJ cancer after neoadjuvant CRT as demonstrated by the CheckMate 577 trial.^10^ In May 2021, the US Food and Drug Administration approved nivolumab for adjuvant treatment in adults with completely resected E/GEJ cancer who received neoadjuvant CRT and had residual pathologic disease after surgery, regardless of PD-L1 expression.^11^ In addition, nivolumab received a Category 1 recommendation as an adjuvant option for E/GEJ cancer after preoperative chemoradiation with complete R0 resection and residual pathologic disease.^4^ More recently, the 2025 American Society of Clinical Oncology annual meeting reported long-term follow-up from CheckMate 577, which confirmed sustained DFS benefit and showed a numerical improvement in OS with adjuvant nivolumab (median OS 51.7 vs 35.3 months), though the difference did not reach statistical significance.^12^ These findings further support the role of adjuvant nivolumab.

Given both US Food and Drug Administration approval and uniform NCCN consensus, early adoption of adjuvant nivolumab was seen in our integrated health system, but little is known of the patient journey leading up to and during adjuvant nivolumab therapy in the real world. Here, the term “patient journey” is used to describe treatment timelines, decision points, and real-world implementation of adjuvant nivolumab. Currently, the literature is sparse in discussing the patient's journey, as well as the clinician's choice as to whether to initiate and/or continue adjuvant nivolumab.

Previous real-world research from our group in the adjuvant setting in patients with esophageal cancer has found that most patients were unable to complete immunotherapy treatment, with reasons including disease progression or side effects, with immunotherapy not resulting in a statistically significant 1-year survival benefit.^13^ This retrospective cohort study aimed to assess the real-world use of adjuvant nivolumab in patients with E/GEJ cancer treated at Kaiser Permanente Northern California (KPNC). By analyzing patient characteristics, survival outcomes, and the patient journey, this study seeks to provide a comprehensive understanding of the benefits and challenges associated with adjuvant nivolumab in a real-world setting. We hypothesized that although most patients would meet eligibility criteria for adjuvant nivolumab based on CheckMate 577 and NCCN guidelines, the actual proportion receiving therapy would be lower due to clinical and patient-level decision-making. We further hypothesized that reasons for treatment omission or discontinuation would vary widely, and that the rate of Combined Positive Score (CPS) testing would be under 50%, reflecting a potential gap in biomarker integration in clinical practice. These findings will contribute to the growing body of evidence supporting the use of immune checkpoint inhibitors in the adjuvant treatment of E/GEJ cancer and may help identify areas for optimization in clinical practice.

## Methods

This was a retrospective, non-interventional cohort study of electronic health record (EHR) data in the KPNC cancer registry. Included patients were diagnosed with E/GEJ cancer and received CRT per the CROSS-trial protocol (carboplatin and pemetrexed), with the majority of patients receiving either 4140 or 4500 centigrays (cGy) of radiation, had their cancer resected, and received adjuvant treatment all at KPNC. The index date was the date of CRT. The index period was December 1, 2020, to August 31, 2023. Baseline data were captured during the 6 months before index and follow-up data were captured for a minimum of 12 months following index, or until death, disenrollment from Health Plan, or last contact date up to October 31, 2024. The study period was June 1, 2020, to October 31, 2024. The datasets generated and/or analyzed during the current study are not publicly available owing to their being the property of Kaiser Foundation Health Plan, Inc.

### Data Source

This study used data from the KPNC cancer registry (meeting standards of Surveillance, Epidemiology, and End-Results), HealthConnect, Kaiser Permanente's EPIC-based EHR, which was established in 2003. KPNC is an integrated health care system serving a diverse population across 18 medical centers and more than 70 outpatient clinic locations across California. KPNC represents the largest private-sector EHR and includes access to medical and pharmacy records from both inpatient and outpatient services. This study was approved by the institutional review board of KPNC under exempt status on October 28, 2024 (IRB #2095829), and individual consent for this retrospective analysis was waived due to minimal risk.

### Patient Selection

Manual record review was conducted to confirm the study eligibility, receipt of nivolumab, reason for not receiving nivolumab if eligible, nivolumab treatment completion, and reason for not completing nivolumab treatment. Patients were included if they were aged at least 18 years at index; were diagnosed with stage II/III E (adenocarcinoma or squamous cell)/GEJ cancer and received CRT within the index period; had evidence of residual disease on surgical pathology (nonpathologic complete response) after neoadjuvant CRT at index; and had an Eastern Cooperative Oncology Group performance score (ECOG PS) 0-1 at index. Patients were excluded if they did not receive CRT. All patients had at least 12 months of follow-up from the index date (CRT completion). Patients who died within 12 months of initiating adjuvant nivolumab were not excluded; their time-to-event outcomes were captured in the analyses.

### Demographics/Outcome Measures

Outcomes and demographic measures collected included patient demographic characteristics (age, sex, race/ethnicity), clinical data (body mass index, smoking history, Charlson Comorbidity Index, Neighborhood Deprivation Index [NDI], insurance type, follow-up time, ECOG PS, tumor location, histology type, PD-L1 status, CPS status, human epidermal growth factor receptor 2 status, recurrence, and recurrence type).

Patient journey outcomes included procedure type, resection (R) status, time from surgery to nivolumab, time from CRT to nivolumab, nivolumab duration, initiation nivolumab dosing frequency, completed nivolumab, reason for noncompletion, progression (distant recurrence, adverse events [AEs]), and time to side effects. Locoregional recurrence rates after neoadjuvant therapy were also identified. Pathologic tumor staging was assessed using the American Joint Committee on Cancer 8th edition ypT classification, which reflects the depth of residual tumor invasion after neoadjuvant therapy.

Clinical outcomes collected included OS, DFS, and distant metastases-free survival (DMFS). OS was defined as time from treatment initiation (nivolumab first dose date) to death date (any cause of death) or last contact date (up to October 31, 2024). DFS was defined as the time from treatment initiation (nivolumab first dose date) to date of disease recurrence, death, or last contact date (up to October 31, 2024), whichever occurred first. DMFS was defined as time from treatment initiation (nivolumab first dose date) to the date of first distant recurrence, death, or last contact date (up to October 31, 2024), whichever occurred first. Stratified exploratory analysis for subgroups including treatment completion status, nodal status, timing to nivolumab initiation, and number of doses and treatment duration was also performed.

### Statistical Analysis

All statistical analyses were performed using SAS, version 9.4. Descriptive statistics were used to describe all categorical and continuous variables. Continuous variables were summarized using means, medians, standard deviations, and interquartile ranges (IQRs). Categorical variables were summarized using frequencies and proportions. The Kaplan-Meier product limit method was used to estimate unadjusted OS, DFS, and DMFS rates. Owing to the small sample size, multivariable regression model was not conducted.

## Results

**Figure 1:**
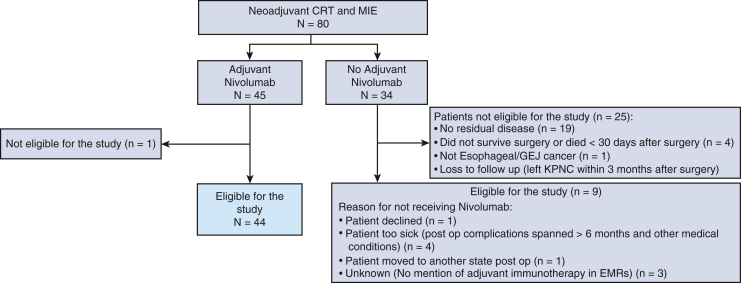
Patient attrition. *CRT*, Chemoradiation therapy; *MIE*, minimally invasive esophagectomy; *E/GEJ*, Esophageal/gastroesophageal junction; *KPNC*, Kaiser Permanente Northern California; *EMR*, electronic medical record.

Of 80 potentially eligible patients, 45 received adjuvant nivolumab, with 44 of those meeting the criteria to be included in this study (the majority of excluded patients had no residual disease) (Figure 1).

### Patient Characteristics

The median age of patients was 66.0 (IQR, 61.5-73.0) years at CRT index date (Table 1). Most patients were male (n = 33, 75%) and White (n = 31, 70.5%). Median follow-up from cancer diagnosis was 26.3 (IQR, 17.3-33.3) months, and from nivolumab first dose 19.1 (IQR, 10.6-25.3) months. Most patients had either Medicare (n = 22, 50%) or commercial (n = 19, 43.2%) insurance. Most patients had a tumor located at the GEJ (n = 24, 54.5%), adenocarcinoma histology (n = 40, 90.9%), and stage III disease (n = 37, 84.1%). Most patients (n = 33, 70.5%), did not receive PDL-1 or CPS testing.

**Table 1:** Patient demographic and clinical characteristics

Variables	Total (N = 44)
Age, y, at CRT index date	
Mean ± SD	65.9 ± 7.6
Min-max	50.0-81.0
Median (IQR)	66.0 (61.5-73.0)
Sex, n (%)	
Female	11 (25%)
Male	33 (75%)
Race/ethnicity, n (%)	
African American	1 (2.3)
Asian/Pacific Islander	6 (13.6)
Hispanic	6 (13.6)
White	31 (70.5)
Smoking history, n (%)	
Current	5 (11.4)
Nonsmoker	17 (38.6)
Former smoker	22 (50.0)
Follow-up time from cancer diagnosis date to death/last contact date, mo	
Mean ± SD	26.0 (9.1)
Min-max	12.8-45.0
Median (IQR)	26.3 (17.3-33.3)
Follow-up time from nivolumab first dose date to death/last contact date, mo	
Mean ± SD	18.9 (9.1)
Min-max	5.1-39.7
Median (IQR)	19.1 (10.6-25.3)
Health insurance, n (%)	
Commercial	19 (43.2)
Medicaid	3 (6.8)
Medicare	22 (50.0)
NDI, quartiles, n (%)	
1 (least deprived)	10 (22.7)
2	13 (29.5)
3	13 (29.5)
4 (most deprived)	8 (18.2)
BMI, kg/m^2^, n (%)	
<25.0	16 (36.4)
25.0–29.9	13 (29.5)
≥30.0	15 (34.1)
BMI, kg/m^2^	
Mean ± SD	28.4 ± 6.3
Min-max	17.3-48.7
Median (IQR)	27.3 (23.8-33.3)
ECOG PS, n (%)	
0	30 (68.2)
1	14 (31.8)
Tumor location, n (%)	
Upper third	1 (2.3)
Middle third	3 (6.8)
Lower third	16 (36.4)
GEJ	24 (54.5)
Histology, n (%)	
Adenocarcinoma, NOS	40 (90.9)
Squamous cell carcinoma, NOS	4 (9.1)
Clinical stage, n (%)	
II	7 (15.9)
III	37 (84.1)
CCI, n (%)	
0-3	14 (31.8)
4-6	11 (25.0)
7+	19 (43.2)
PD-L1 status,∗n (%)	
Untested	31 (70.5)
Negative	10 (22.7)
Positive	3 (6.8)
CPS status,∗n (%)	
1-10	8 (18.2)
>10	3 (6.8)
Untested	31 (70.5)
HER2 status, n (%)	
Untested	20 (45.5)
Negative	13 (29.5)
Positive	11 (25.0)
Recurrence, n (%)	
No	24 (54.6)
Yes	20 (45.5)
Recurrence type, n (%)	
Local	1 (5.0)
Distant	19 (95.0)

*CRT*, Chemoradiation therapy; *SD*, standard deviation; *IQR*, interquartile range; *NDI*, Neighborhood Deprivation Index; *BMI*, body mass index; *ECOG PS*, Eastern Cooperative Oncology Group performance score; *GEJ*, gastroesophageal junction; *NOS*, not otherwise specified; *CCI*, Charlson Comorbidity Index; *PD-L1*, programmed death-ligand 1; *CPS*, Combined Positive Score; *HER2*, human epidermal growth factor receptor 2.

∗ PD-L1 and CPS were not routinely tested in patients with stage II/III E/GEJ.

### Patient Journey

All patients received minimally invasive esophagectomy (MIE), with 100% also achieving an R0 resection status (indicating no residual tumor on pathology) (Table 2). The time from surgery to the initiation of nivolumab treatment varied, with a median of 53.5 days (IQR, 46.0-75.5 days). The median time from CRT to nivolumab initiation was 165 days (IQR, 138.5-194 days). The median duration of nivolumab treatment was 322 days (IQR, 148.5-364 days). Initial dosing frequency of nivolumab was predominantly every 2 weeks for 77.3% of patients, and 22.7% every 4 weeks. Completion of the nivolumab treatment regimen was achieved by 52.3% of patients, while 47.7% did not complete treatment. Treatment completion by initial dosing frequency did not differ (Tables E1 and E2). Of these, 13 (61.9%) patients did not complete treatment owing to progression (distant recurrence), with 8 (38.1%) not completing owing to AEs (the most common [n = 3] being pneumonitis). The median time to development of an AE was 140 days (IQR 85.5-199.5).

**Table 2:** Patient journey and adverse events

Variables	Total (N = 44)
Procedure type, n (%): MIE	44 (100)
R status, n (%): R0	44 (100)
Time from surgery to nivolumab, d	
Mean ± SD	67.5 ± 42.6
Min-max	27-267
Median (IQR)	53.5 (46.0-75.5)
Time from CRT to nivolumab, d	
Mean ± SD	171.4 ± 47.7
Min-max	112-363
Median (IQR)	165 (138.5-194)
Nivolumab duration, d	
Mean ± SD	293.7 ± 1 86.3
Min-max	0-1032
Median (IQR)	322 (148.5-364)
Initial dosing frequency, wk, n (%)	
Every 2 wk	34 (77.3)
Every 4 wk	10 (22.7)
Nivolumab complete, n (%)	
No	21 (47.7)
Yes	23 (52.3)
Reason for not complete, n (%)	
Progression, distant recurrence	13 (61.9)
AEs, all grades	8 (38.1)
Progression	
Local recurrence	0
Distant recurrence	13
AE type	
Colitis	2
Dysgeusia, mouth sores	1
Hepatitis, renal insufficiency, anorexia, fatigue	1
Immune-related arthritis	1
Pneumonitis	3
Time to side effect, d, median (IQR)	140 (85.5-199.5)
Time to side effect, d	
Month 1	1
Month 1-2	1
Month 3-6	4
>6 mo	2

*MIE*, Minimally invasive esophagectomy; *R*, resection; *SD*, standard deviation; *IQR*, interquartile range; *CRT*, chemoradiation therapy; *AE*, adverse event.

### Recurrence

Of 44 patients, 20 (45.5%) developed recurrence. Recurrence occurred in 7 of 21 (33.3%) patients with ypT1/T2 tumors and 13 of 23 (56.5%) with ypT3/T4 tumors (*P* = .123). Locoregional recurrence occurred in 1 patient (2.3%), and distant recurrence occurred in 19 patients (43.2%). Among ypT1/T2 patients with recurrence, 1 (14.3%) was locoregional and 6 (85.7%) were distant; all recurrences in the ypT3/T4 group were distant (*P* = .350) (Table E3).

### Primary Outcome Results

#### Clinical outcomes

**Figure 2:**
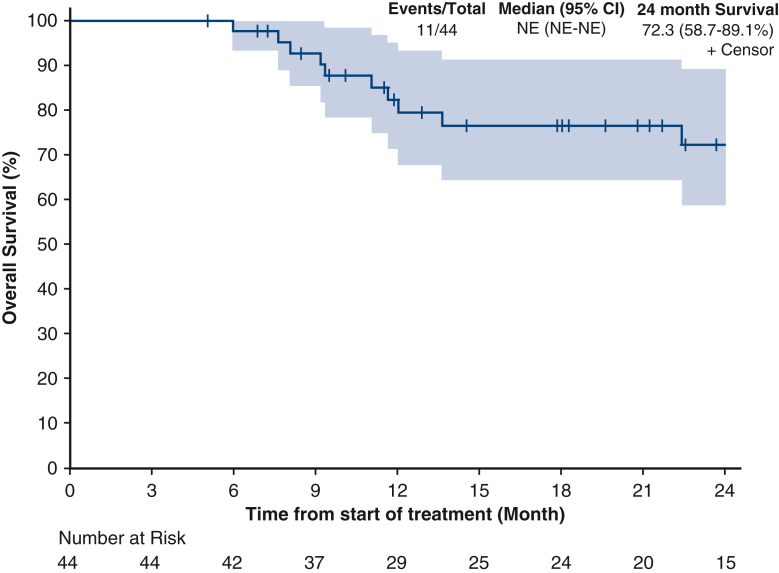
Overall survival in 12 months (NE [CI, NE-NE]) and 24 months (72.3 [CI, 58.7%-89.1%]). *NE*, Not estimable; *CI*, confidence interval.

**Figure 3:**
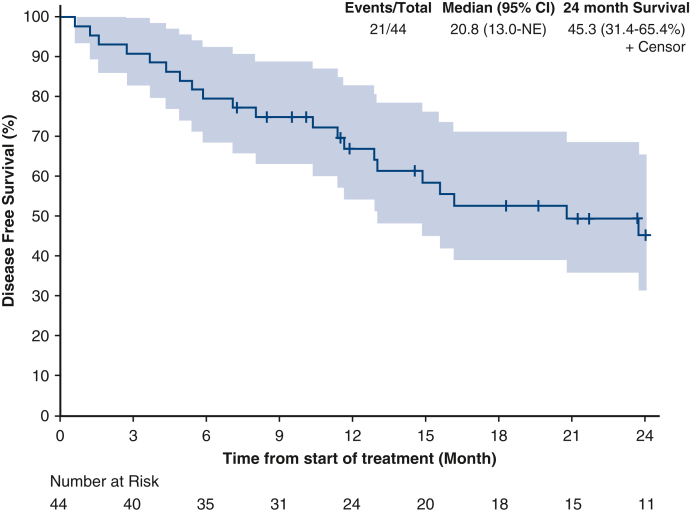
Disease-free survival in 12 months (20.8 [CI, 13.0-NE]) and 24 months (45.3 [CI, 31.4%-65.4%]). *NE*, Not estimable; *CI*, confidence interval.

**Figure 4:**
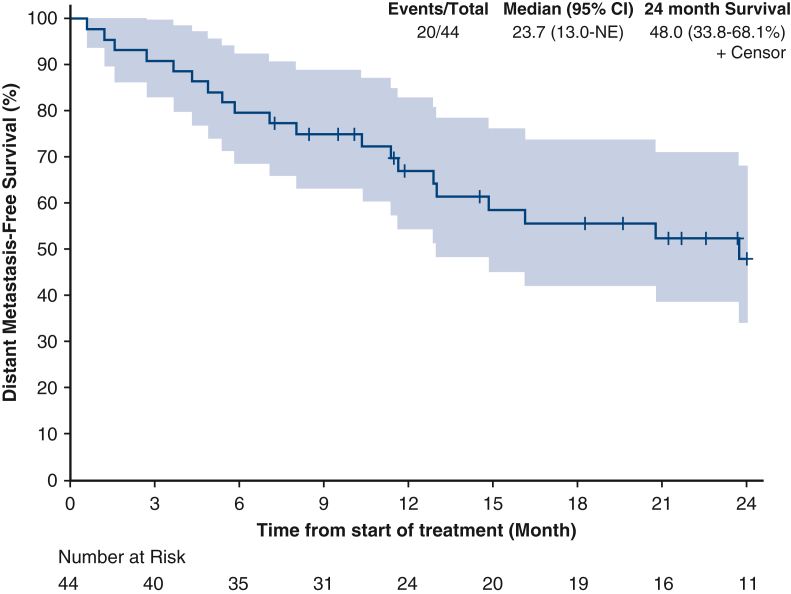
Distant metastases-free survival in 12 months (23.7 [CI, 13.0-NE]) and 24 months (48 [CI, 33.8%-68.1%]). *NE*, Not estimable; *CI*, confidence interval.

Median OS was not reached (Figure 2). The 24-month OS rate was 72.3% (95% confidence interval [CI], 58.7%-‍89.1%). Median DFS was 20.8 months (95% CI, 13-not estimable [NE]) (Figure 3). The 24-month DFS rate was 45.3% (95% CI, 31.4%-65.4%). Median DMFS was 23.7 months (95% CI, 13.0-NE) (Figure 4). The 24-month DMFS rate was 48.0% (95% CI, 33.8%-68.1%).

## Stratified Analysis

### OS and DFS by Nivolumab Treatment Completion Status

**Figure E1:**
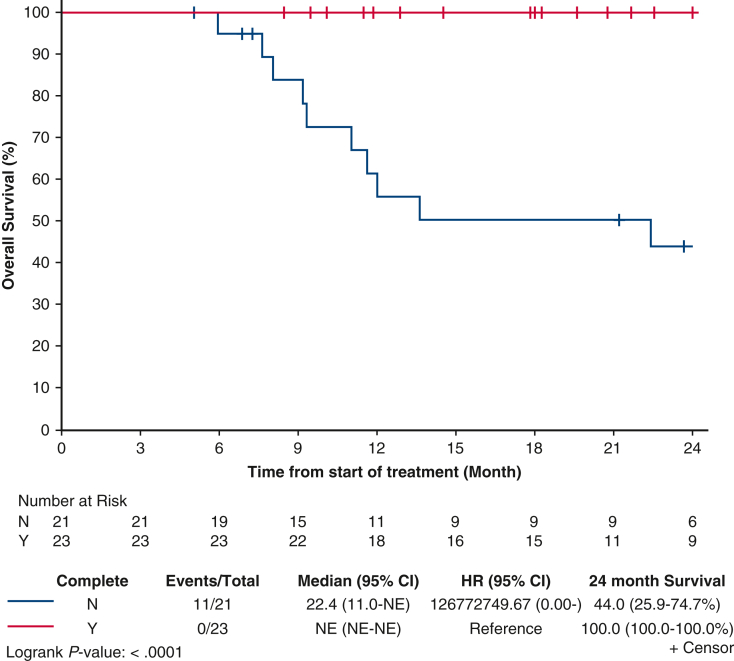
Overall survival stratified by nivolumab treatment completion in 12 months (NE [CI, NE-NE]) and 24 months (100 [CI, 100.0%-100.0%]). *NE*, Not estimable; *CI*, confidence interval; *HR*, hazard ratio.

**Figure E2:**
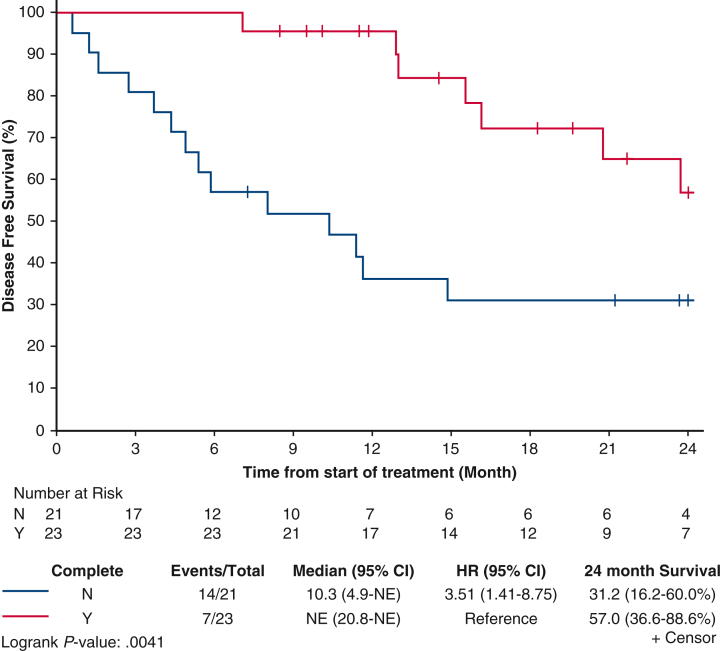
Disease-free survival stratified by nivolumab treatment completion 12 months (NE [CI, 20.8-NE]) and 24 months (57 [CI, 36.6%-88.6%]). *NE*, Not estimable; *CI*, confidence interval; *HR*, hazard ratio.

Median OS for those who did not complete treatment was 22.4 months (95% CI, 11.0-NE), and median OS for those who completed treatment was not reached (Figure E1). The 24-month OS was 44% (95% CI, 26%-74.7%) in the incomplete group versus 100% in the treatment complete group (log-rank *P* < .001). Median DFS was 10.3 months (95% CI, 4.9–NE) in the incomplete group and not reached in the complete group (Figure E2). The 24-month DFS was 31.2% (95% CI, 16.2%-60.0%) in those who did not complete treatment, compared with 57.0% (95% CI, 36.6%-88.6%) in those who did (log-rank *P* = .0041).

### OS and DFS by Nodal Status

**Figure E3:**
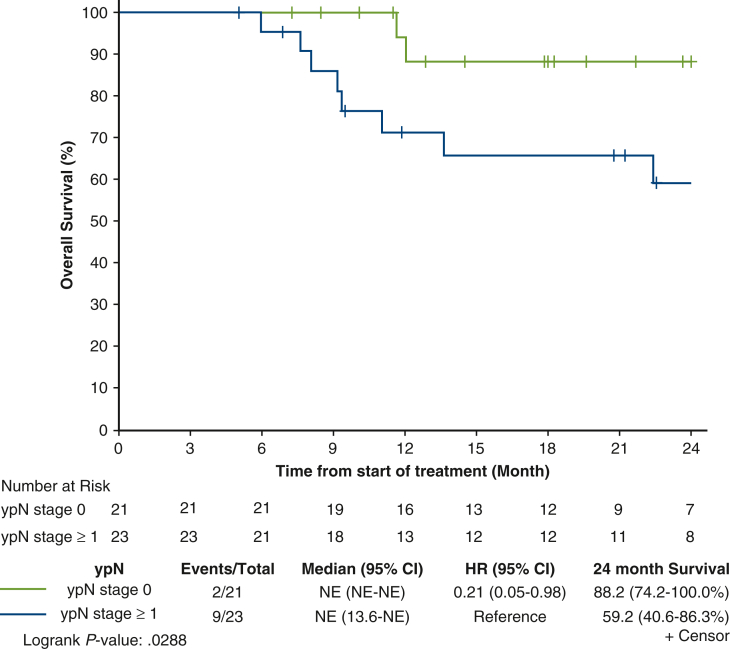
Overall survival stratified by ypN stage with ypN0 12 months (NE [CI, NE-NE]) and 24 months (88.2 [CI, 74.2%-100%]) and ypN greater than 1, 12 months (NE [CI, 13.6-NE]) and 24 months (59.2 [CI, 40.6%-86.3%]). *NE*, Not estimable; *CI*, confidence interval; *HR*, hazard ratio.

**Figure E4:**
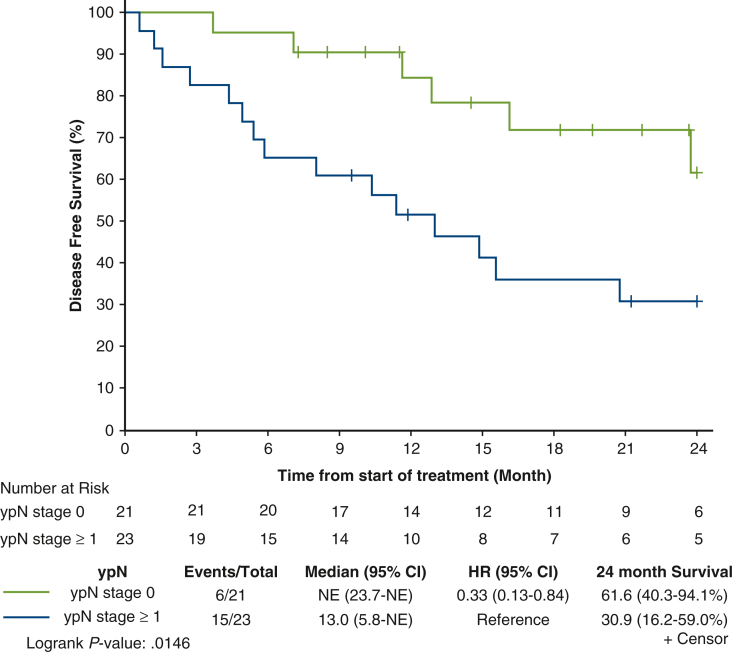
Disease-free survival stratified by ypN stage with ypN0 12 months (NE [CI, 23.7-NE]) and 24 months (61.6 [CI, 40.3%-94.1%]) and ypN greater than 1, 12 months (13.0 [CI, 5.8-NE]) and 24 months (30.9 [CI, 16.2%-59.0%]). *NE*, Not estimable; *CI*, confidence interval; *HR*, hazard ratio.

For ypN status, median OS was not reached in either group (Figure E3). The 24-month OS was 88.2% (95% CI, 74.2%-100%) for patients who were ypN0 and 59.2% (95% CI, 40.6%- 86.3%) for ypN + patients (log-rank *P* = .0288). Median DFS was not reached for patients who were ypN0, and for ypN+ was 13.0 months (95% CI, 8.8-NE) (Figure E4). The 24-month DFS for ypN0 was 61.6% (95% CI, 40.3%-94.1%) and 30.9% (95% CI, 16.2%-59.0%) for ypN+ (log-rank *P* = .0146).

### OS and DFS by Timing to Nivolumab Initiation

**Figure E5:**
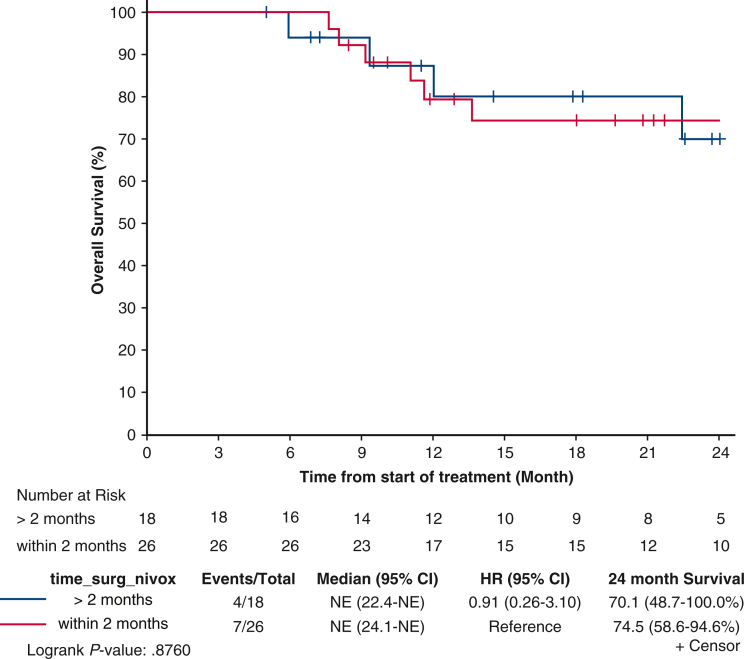
Overall survival stratified by time from surgery to nivolumab initiation within 2 months (12 months NE [CI, 24.1-NE] and 24 months 74.5 [CI, 58.6%-94.6%]) versus greater than 2 months (12 months NE [CI, 22.4-NE] and 24 months 70.1 [CI, 48.7%-100.0%]). *NE*, Not estimable; *CI*, confidence interval; *HR*, hazard ratio.

**Figure E6:**
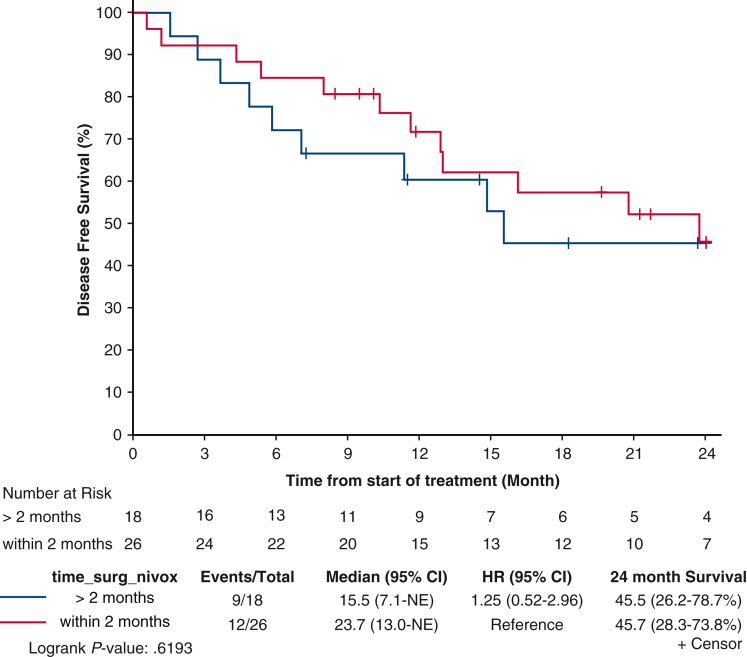
Disease-free survival stratified by time from surgery to nivolumab initiation within 2 months (12 months NE [CI, 24.1-NE] and 24 months 74.5 [CI, 58.6%-94.6%] vs greater than 2 months (12 months 15.5 [CI, 7.1-NE] and 24 months 45.5 [CI, 26.2%-78.7%]). *NE*, Not estimable; *CI*, confidence interval; *HR*, hazard ratio.

Among the 44 patients, 26 (59%) initiated nivolumab within 2 months, and 18 (41%) initiated after 2 months. Median OS was not reached for either group (Figure E5). 24-month OS was 74.5% (95% CI, 58.6%-94.6%) in the within 2 months group, and 70.1% (95% CI, 48.7%-100.0%) in the after 2 months group (log-rank *P* = .8760). Median DFS was 23.7 months (95% CI, 13.0-NE) in the within 2 months group, and 15.5 months (95% CI, 7.1-NE) in the after 2 months group. 24-month DFS was 45.7% (95% CI, 28.3%-73.8%) in the within 2 months group and 45.5% (95% CI, 26.2%-78.7%) in the after 2 months group (log-rank *P* = .6193) (Figure E6).

### OS and DFS by Nivolumab Total Number of Doses and Treatment Duration

**Figure E7:**
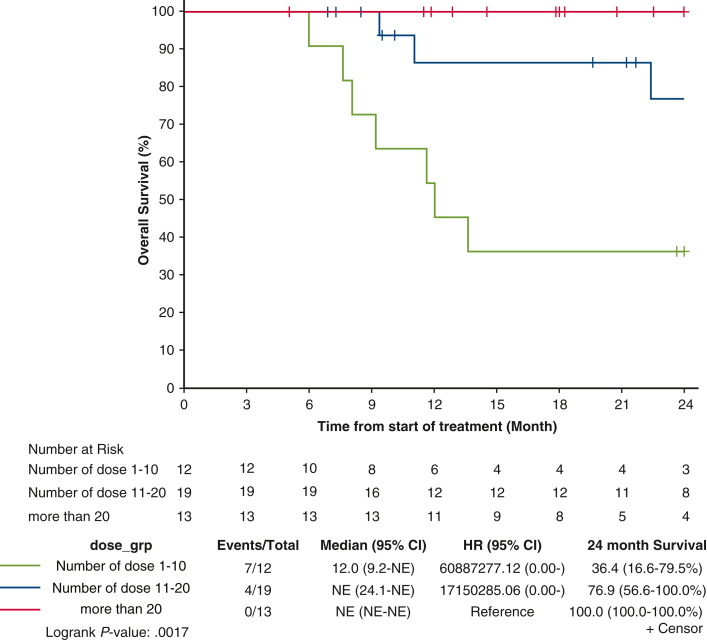
Overall survival stratified by number of nivolumab doses with 1-10 doses (12 months 12.0 [CI, 9.2-NE] and 24 months 36.4 [CI, 16.6%-79.5%]), 11-20 doses (12 months NE [CI, 24.1-NE] and 24 months 76.9 [CI, 56.6%-100%]), and more than 20 doses (12 months NE [CI, NE-NE] and 24 months 100.0 [CI, 100.0%-100.0%]). *NE*, Not estimable; *CI*, confidence interval; *HR*, hazard ratio.

**Figure E8:**
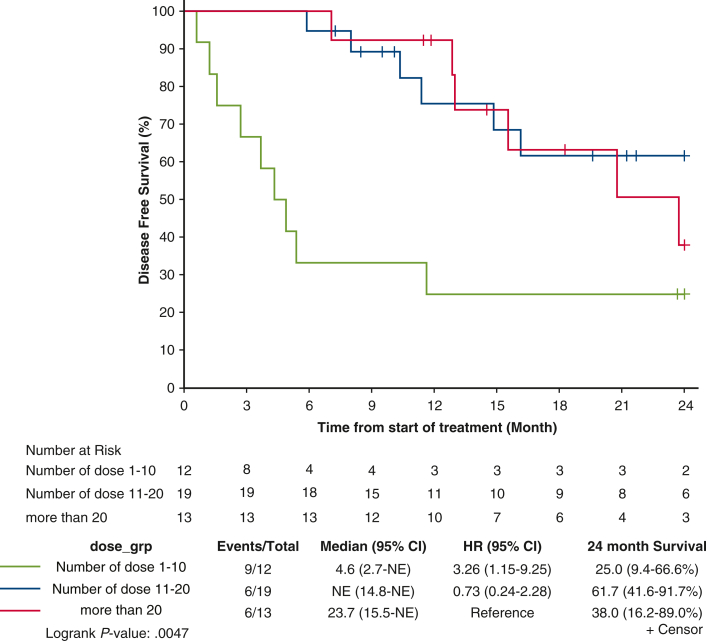
Disease-free survival stratified by number of nivolumab doses with 1-10 doses (12 months 4.6 [CI, 2.7-NE] and 24 months 25.0 [CI, 9.4%-66.6%]), 11-20 doses (12 months NE [CI, 14.8-NE] and 24 months 61.7 [CI, 41.6%-91.7%]), and more than 20 doses (12 months 23.7 [CI, 15.5-NE] and 24 months 38 [CI, 16.2%-89.0%]). *NE*, Not estimable; *CI*, confidence interval; *HR*, hazard ratio.

**Figure E9:**
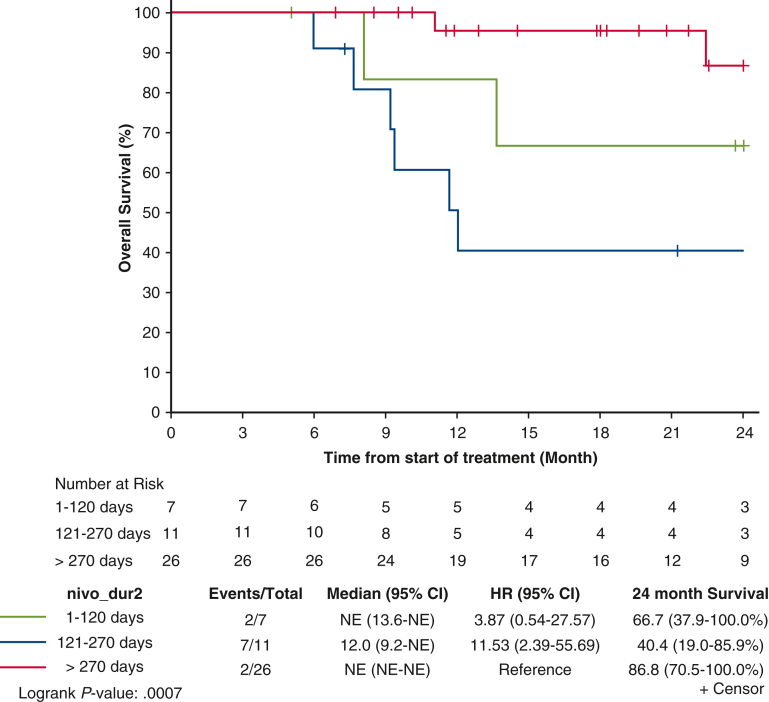
Overall survival stratified by nivolumab treatment duration with 1-120 days (12 months NE [CI, 13.6-NE] and 24 months 66.7 [CI, 37.9%-100.0%]), 121-270 days (12 months 12.0 [CI, 9.2-NE] and 24 months 40.4 [CI, 19.0%-85.9%]), and greater than 270 days (12 month NE [CI, NE-NE] and 24 months 86.8 [CI, 70.5%-100.0%]). *NE*, Not estimable; *CI*, confidence interval; *HR*, hazard ratio.

**Figure E10:**
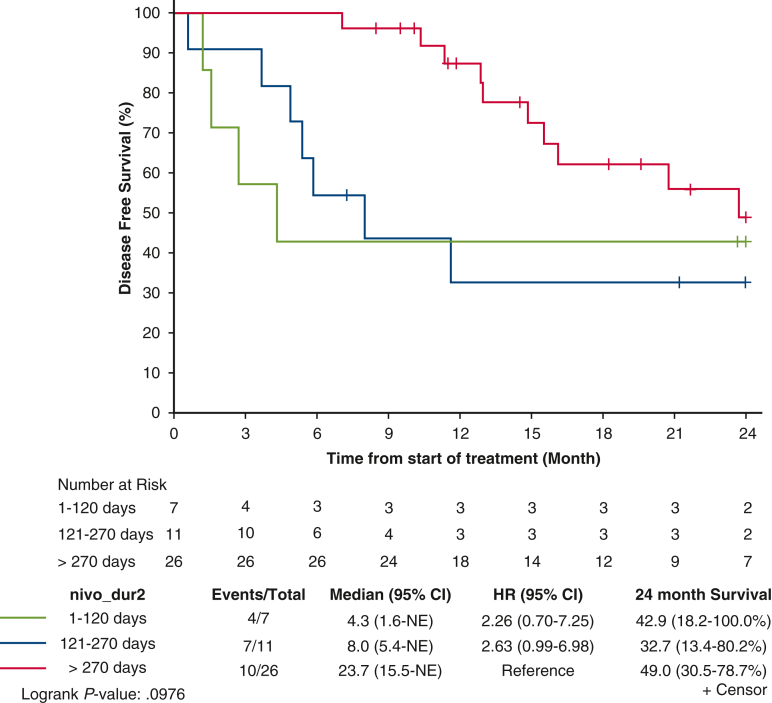
Disease-free survival stratified by nivolumab treatment duration with 1-120 days (12 months 4.3 [CI, 1.6-NE] and 24 months 42.9 [CI, 18.2%-100.0%]), 121-270 days (12 months 8.0 [CI, 5.4-NE] and 24 months 32.7 [CI, 13.4%-80.2%]), and greater than 270 days (12 month 23.7 [CI, 15.5-NE] and 24 months 49.0 [CI, 30.5%-78.7%]). *NE*, Not estimable; *CI*, confidence interval; *HR*, hazard ratio.

Patients were categorized into tertiles to analyze the effect of number of doses and treatment duration on outcomes (Figure E7, Figure E8, Figure E9, Figure E10). The median OS in the 1-10 doses group was 12.0 months (95% CI, 9.2-NE). Median OS was not reached in the other groups (11-20 doses and >20 doses) (Figure E7). The 24-month survival in the 1-10 doses group was 36.4% (95% CI, 16.6%-79.5%), in the 11-20 doses group 76.9% (95% CI, 56.6%-100%), and in the >20 doses group 100% (100%-100%) (log-rank *P* = .0017). Median DFS in the 1-10 doses group was 4.6 months (95% CI, 2.7-NE), it was not reached in the 11-20 doses group and in the >20 doses group, it was 23.7 months (15.5-NE) (Figure E8). The 24-month survival in the 1-10 doses group was 25% (95% CI, 9.4%-66.6%), in the 11-20 doses group, 61.7% (95% CI, 41.6%-91.7%), and in the >20 doses group 38% (95% CI, 16.2%-89.0%) (log-rank *P* = .0047).

In the analysis to evaluate the effect of treatment duration, the median OS in 121 to 270 days group was 12.0 months (95% CI, 9.2-NE). Median OS was not reached in the 1 to 120 days or the >270 days groups (Figure E9). The 24-month survival in the 1-120 days group was 66.7% (95% CI, 37.9%-100%), in the 121 to 270 days group it was 40.4% (95% CI, 19.0%-85.9%), and in the >270 days group, it was 86.8% (95% CI, 70.5%-100%). Median DFS in the 1-120 days group was 4.3 months (95% CI, 1.6-NE), in the 121 to 270 days group was 8.0 months (95% CI, 5.4- NE), and in the >270 days was 23.7 months (95% CI, 15.5-NE) (Figure E10). The 24-month survival in the 1-120 days group was 42.9% (95% CI, 18.2%-100%), in the 121-270 days group 32.7% (95% CI, 13.4%-80.2%), and in the >270 days group, 49.0% (95% CI, 30.5%-78.7%) (log-rank *P* = .0976).

## Discussion

This retrospective cohort study provides valuable insights into the real-world use of adjuvant nivolumab in patients with E/GEJ cancer who received CRT and subsequent MIE. The study's findings suggest that adjuvant nivolumab may offer clinical benefits, although several areas for improvement remain, including standardization of treatment initiation times and improving the proportion of patients discontinuing treatment owing to progression or AEs.

The patient cohort was predominantly male and White, with a median age of 66 years. The percentage of patients in minority groups was higher than typically observed in clinical trials, with clinical trials including 3% to 6% patients with Hispanic ethnicity, and 2% Asian American.^13^, ^14^, ^15^ This study included 13% of patients with Hispanic ethnicity; California has a greater-than-average (for the United States) population of Hispanic residents (40.4% Hispanic or Latino), which may account for this, and 13% Asian American.^16^ Our cohort also included patients from a broad range of socioeconomic backgrounds, as demonstrated by the variability in NDI scores. This is meaningful, given growing evidence that lower socioeconomic status is associated with reduced participation in cancer clinical trials, contributing to disparities in access to emerging therapies.^17^ Nonetheless, future studies may wish to focus on increased diversity of patient population to ensure results are relevant to populations affected with E/GEJ cancers.

The median OS was not reached in this study, and the 24-month OS rate was 72.3%, suggesting that nivolumab may extend survival in this high-risk population. However, nearly one half of the patients in our cohort did not complete the full course of therapy, most commonly due to disease progression. To further evaluate the relationship between treatment duration and outcomes, we stratified both OS and DFS by nivolumab treatment completion status. Patients who completed therapy had improved 24-month OS and DFS compared with those who did not. These results suggest that treatment completion may be associated with improved disease control and survival. However, this analysis is limited by small samples sizes and the potential for survivor bias, as the ability to complete therapy may reflect less aggressive disease or better baseline performance status.

The overall median DFS in our cohort was 20.8 months, and the 24-month DFS rate was 45.3%, indicating that although nivolumab delays disease recurrence, a proportion of patients still experience relapse within 2 years. Notably, DFS in this study was similar to that observed in the CheckMate-577 clinical trial (22.4 months, 95% CI 16.6-34.0, n = 532), suggesting that real-world results are consistent with those in the trial setting.^10^ Similarly, the median DMFS and the 24-month DMFS rate track closely with outcomes reported in the pivotal trial.^10^ These findings are further supported by the long-term follow-up data (median follow-up of 78.3 months) from CheckMate 577 presented at the 2025 American Society of Clinical Oncology Annual Meeting, which demonstrated sustained DFS benefit with adjuvant nivolumab (27.3 months; 95% CI, 21.4-36.0, n = 532).^12^

Given the prognostic significance of residual nodal disease in esophageal and GEJ cancers, we also conducted an exploratory analysis stratified by ypN status. Consistent with findings from the CheckMate 577 trial, we observed that patients with ypN + disease had worse OS and DFS compared with those with ypN0 status. Although limited by small sample sizes, these trends highlight the importance of nodal involvement in risk stratification.

Regarding disease recurrence in our cohort, 45.5% of patients experienced disease recurrence, with 95% of those being distant metastases. Locoregional recurrence was rare, occurring in only one patient (2.3%), a case limited to the ypT1/T2 group. All recurrences in patients with ypT3/T4 disease were distant. In the CheckMate 577 trial, locoregional recurrence rates were 12% in the nivolumab arm and 17% in the placebo arm, while distant recurrence was reduced from 39% to 29% with adjuvant therapy. Compared with these trial results, our cohort showed fewer locoregional events, likely reflecting the small sample size.

Compared with the CheckMate 577 trial, our cohort included a larger proportion of GEJ tumors and adenocarcinoma, whereas the trial included a larger proportion of esophageal tumors and squamous cell carcinoma. Subgroup analyses from CheckMate 577 demonstrated that the benefit of adjuvant nivolumab varied by tumor location and histology. The hazard ratio (HR) for disease recurrence or death favored nivolumab more in patients with esophageal cancer (HR, 0.61) than in those with GEJ cancer (HR, 0.87). Among patients who received nivolumab, the median DFS was similar between esophageal and GEJ subgroups. However, in the placebo arm, median DFS was longer in patients with GEJ cancer than in those with esophageal cancer. Furthermore, adenocarcinoma had an intermediate HR of 0.75, with a median DFS improvement of 8.3 months compared with placebo. These patterns highlight that tumor location and histologic subtype may influence immunotherapy outcomes, and may help explain differences in recurrence observed between our real-world cohort and the trial population.

The patient journey and treatment adherence results demonstrate that all patients achieved an R0 resection status, underscoring the effectiveness of MIE in this cohort. This is likely due to KPNC being an integrated health system consisting of 21 medical centers with 4 centers of excellence where MIEs are performed. These centers of excellence are high volume centers each performing over 20 minimally invasive esophagectomies per year, with recent literature from our group showing superior R0 rates, nodal harvest, morbidity and mortality rates.^18^, ^19^, ^20^ Further, KPNC typically facilitates efficient coordination of care, contributing to timely initiation of adjuvant therapy. Nevertheless, we observed variability in the time from surgery to nivolumab initiation and from CRT to initiation, suggesting a lack of standardization in timing. In an exploratory analysis, we found no difference in OS or DFS when stratified by whether nivolumab was initiated within 2 months or >2 months after surgery, indicating that differences in timing may not substantially affect outcomes. However, in the CheckMate 577 trial, the magnitude of benefit in DFS was greater in patients who started nivolumab ≥10 weeks after surgery.^10^ These findings reinforce the need for further studies to define optimal timing windows and to develop standardized guidelines for adjuvant nivolumab initiation across diverse care settings.

The median duration of nivolumab treatment was 322 days, but nearly one half of the patients did not complete the regimen. Similarly, in the CheckMate 577 trial, 43% of patients completed the planned 1-year regimen. Reported reasons for discontinuation included progressive disease (28%), treatment-related toxicities (11%), AEs not attributed to treatment (3%), patient decision (8%), and other causes (2%). A comparative analysis in which we evaluated treatment completion by initial nivolumab dosing frequency (every 2 [Q2] vs every 4 [Q4] week) was not statistically significant. The findings from the trial, as well as from our cohort, highlight the challenges of maintaining long-term immunotherapy in patients with E/GEJ cancer, a population that often demonstrates limited tolerance to adjuvant treatment.^13^^,^^21^ Our study aimed to describe the frequency, type, and timing of AEs to help clinicians better understand patterns of tolerability in routine practice and to help patients understand their likelihood of successfully completing treatment. The sample size of this study, gives a more thorough understanding of these estimates, with previous studies, outside of the clinical trial, presenting further limited patient numbers (for example, in a previous study only 3 of 17 [18%] patients completed treatment).^13^

The study identified pneumonitis as the most common AE leading to treatment discontinuation, with a median time to side effects of 140 days. Although this finding underscores the importance of vigilant monitoring and supportive care, our study was not designed to assess whether earlier surveillance or intervention could have mitigated toxicity. Our study focused on characterizing AEs and their timing, and did not include assessment of predictors of toxicity or modeling of baseline characteristics associated with treatment discontinuation. Nonetheless, our findings highlight the importance of monitoring and proactive management of AEs to ensure patients can complete the intended course of nivolumab. Important to note is that we do not think the greater pneumonitis rate is a result of KPNC radiation doses, but rather the multifactorial nature of subjectivity of pneumonitis diagnoses by provider clinical judgement and the value that this real-world study adds to the literature in that our data highlights real world data that may be slightly different than what is reported in actual highly regulated clinical trials.

In an exploratory analysis, patients who received more than 20 doses or remained on treatment for greater than 9 months had improved OS and DFS compared with those with shorter treatment durations. However, this study was not powered to evaluate the efficacy of abbreviated treatment courses, and these findings should be interpreted with caution. Furthermore, all patients in our cohort met ECOG 0-1 criteria at baseline, yet many were unable to tolerate prolonged therapy, highlighting the inadequacy of performance status alone in identifying patients likely to benefit from adjuvant immunotherapy. Currently, there are no validated clinical or molecular tools that reliably predict treatment benefit or tolerability in this setting. PD-L1 status was not a requirement in CheckMate 577 trial and was inconsistently documented in our cohort. Further, CPS testing was notably less than 50%, reflecting a potential gap in biomarker integration in clinical practice. Future studies should explore the role of molecular markers and immune profiling on treatment response. Identifying patients for whom additional systemic therapy is both feasible and beneficial is essential to minimizing toxicity and optimizing outcomes in the real-world setting.

This study had several strengths and limitations. Strengths include the use of real-world data, comprehensive follow-up, and detailed patient journey documentation, because KPNC encompasses 4.6 million members of the 12.7 million Kaiser Permanente members nationwide. To our knowledge this is the largest cohort study to date of patients with E/GEJ cancer receiving adjuvant nivolumab in a real-world setting, giving clinicians a valuable source of information to aid treatment choice and understand how a patient may experience their treatment journey. However, limitations such as the retrospective design, small sample size limiting multivariable analysis to limit bias and effect modification, lack of a control group, and variability in treatment timelines must be acknowledged. A subgroup analysis by age, race/ethnicity, sex, and NDI yielded no statistically significant differences in DFS, likely given the small sample size. Further, these study results may not be generalized to the overall US population, as all participants were treated within a single integrated health system. In addition, as a retrospective EHR analysis with chart reviews, there is a potential for missing, incomplete, or inaccurate data, which may introduce bias. The study also did not include detailed perioperative complication data (anastomotic leaks or surgical morbidity), which may influence feasibility or timing of adjuvant therapy. Furthermore, we did not assess quality of life (QOL) outcomes or patient-reported measures, because this was not within the scope of our institutional review board−approved aims and KPNC does not routinely collect structured QOL metrics. Future prospective studies incorporating structured QOL assessments are warranted to complement these real-world findings.

## Conclusions

This study demonstrated the benefits of adjuvant nivolumab in improving survival outcomes for patients with E/GEJ cancer after CRT and MIE, demonstrating survival and safety benefits in line with those observed in clinical studies. The high rates of R0 resection and the promising OS, DFS, and DMFS rates suggest the effectiveness of this treatment approach. Future prospective studies are warranted to explore strategies to mitigate AEs and improve treatment adherence. Future research would also benefit from understanding the relationship between dosing duration of nivolumab and corresponding OS, DFS, and DMFS.

## Conflict of Interest Statement

T.W., G.O., L.R., and R.V. are employees of BMS. All other authors reported no conflicts of interest.

The *Journal* policy requires editors and reviewers to disclose conflicts of interest and to decline handling or reviewing manuscripts for which they may have a conflict of interest. The editors and reviewers of this article have no conflicts of interest.

## Figures and Tables

**Table E1:** Treatment completion by initial nivolumab dosing frequency

Variables	Total (N = 44)	Initial dosing frequency Q2W (n = 34)	Initial dosing frequency Q4W (n = 10)	*P* value
Complete				.287
No	21 (47.7)	18 (52.9)	3 (30.0)	
Yes	23 (52.3)	16 (47.1)	7 (70.0)	

*Q2W*, Once every 2 weeks; *Q4W*, once every 4 weeks.

**Table E2:** Treatment completion and reason for no completion by initial nivolumab dosing frequency

Variables	Total (N = 44)	Complete (n = 23)	Not complete due to progression (n = 13)	Not complete due to adverse event (n = 8)	*P* value
Initial dosing frequency					
Q2	34 (77.3)	16 (69.6)	10 (76.9)	8 (100)	.222
Q4	10 (22.7)	7 (30.4)	3 (23.1)	0	

Q2 = Once every 2 weeks; Q4 = once every 4 weeks.

**Table E3:** Disease recurrence by ypT1/T2 versus ypT3/T4

Variables	Total (N = 44)	ypT1/T2 (n = 21)	ypT3/T4 (n = 23)	*P* value
Recurrence				.123
No	24 (54.5)	14 (66.7)	10 (43.5)	
Yes	20 (45.5)	7 (33.3)	13 (56.5)	
Type				.350
Locoregional	1 (5.0)	1 (14.3)	0	
Distant	19 (95.0)	6 (85.7)	13 (100)	
